# Adherence to the Mediterranean diet and its effects on the onset and progression of diabetic nephropathy: a systematic review

**DOI:** 10.1080/0886022X.2026.2685372

**Published:** 2026-07-27

**Authors:** Ioannis Sykaras, Maria Kantilafti, Stavri Chrysostomou

**Affiliations:** Department of Life Sciences, European University of Cyprus, Nicosia, Cyprus

**Keywords:** Mediterranean diet, diabetes, diabetic nephropathy, eGFR, proteinuria, microvascular complications

## Abstract

**Background:**

Diabetic nephropathy (DN) is the primary microvascular complication of diabetes mellitus (DM), contributing to chronic kidney disease, and end-stage renal failure. More than 247,000 individuals develop renal failure due to DM. Mediterranean diet (MD) associated with cardiometabolic benefits and potential renal protective effects, however, its relationship on the onset and progression of DN remains unclear.

**Objective:**

To examine the association between adherence to the MD and the onset and progression of DN.

**Methods:**

This systematic review was conducted in accordance with PRISMA 2020 guidelines. A comprehensive search was performed in PUBMED, EBSCO host and SCOPUS databases. Studies including adults with hyperglycemia, DM, or DN, regardless of comorbidities, examining adherence to the MD and renal outcomes. Methodological integrity was evaluated using the Newcastle-Ottawa Scale (NOS) and Jadad Scale. Due to heterogeneity across studies, results were synthesized narratively.

**Results:**

Six studies met the inclusion criteria. Most studies demonstrated that higher adherence to the MD was associated with a lower risk or odds of DN, particularly among individuals with DM. Reported associations ranged from 21% to 86%, depending on adherence level and study design. Two studies found no statistically significant associations (*p* > 0.05).

**Limitations:**

The small number of studies, their predominantly observational design, and geographic concentration limit the generalizability and preclude causal inference.

**Conclusion:**

Higher adherence to the MD appears to be associated with a lower risk of DN; however, current evidence remains limited and insufficient to draw conclusions regarding causality or disease progression. Further high-quality studies are required.

## PLAIN LANGUAGE SUMMARY

DN is one of the most common complications of diabetes and a major cause of chronic kidney disease worldwide. This systematic review examined whether following a Mediterranean diet (MD) may help reduce the risk or progression of diabetic nephropathy. Overall, the findings suggest that greater adherence to the MD, characterized by higher intake of fruits, vegetables, legumes, whole grains, fish, nuts, and olive oil, may be associated with lower risk of developing diabetic nephropathy in individuals with diabetes. Potential benefits may be related to improvements in blood glucose control, blood pressure, body weight, and inflammation. However, evidence regarding the progression of established kidney disease remains limited and inconsistent. Further large-scale longitudinal studies are needed to better clarify the long-term renal effects of the MD in diabetic populations.

## KEY MESSAGES

1. What is known: Diabetic nephropathy is a major complication of diabetes, and dietary patterns such as the Mediterranean diet have been associated with improved cardiometabolic health and kidney function.
2. What this study adds: This systematic review suggests that greater adherence to the Mediterranean diet may be associated with a lower risk of incident diabetic nephropathy, although evidence regarding disease progression remains limited.
3. Potential impact: Promoting adherence to the Mediterranean diet may represent a supportive nutritional strategy for reducing renal risk and improving metabolic health in individuals with diabetes.

## Introduction

Diabetic nephropathy (DN) is the leading cause of end-stage renal disease (ESRD) worldwide. It arises primarily from complications associated with chronic hyperglycemia and elevated blood pressure [1–4]. The glomerular filtration barrier is normally unable to retain high molecular weight proteins (e.g. albumin), leading to their leakage into Bowman’s capsule and eventual excretion in the urine. The detection of microalbuminuria is considered an early marker of DN and is often accompanied by an increase in blood pressure. It is important to note that CKD in the context of diabetes and DN are not synonymous. Specifically, CKD in individuals with diabetes is a multifactorial condition that may result from several factors, including hypertension, drug-induced nephrotoxicity, and infections. In contrast, DN refers specifically to renal impairment attributable to diabetes as the primary causative factor [5]. Notably, DN accounts for approximately 4 million deaths annually [4]. According to data from the National Health and Nutrition Examination Survey (NHANES), high mortality rates are strongly associated with the coexistence of diabetes and kidney disease [6].

Among the primary determinants of DN, persistent hyperglycemia and hypertension are of paramount importance. Chronic hyperglycemia can lead to structural and functional damage to the renal microvasculature, increasing the risk of CKD, and, ultimately, progression to renal failure. The advanced stages are also strongly associated with an increased risk of cardiovascular disease [1,2,7,8]. Both factors (hyperglycemia and hypertension), however, can be significantly influenced through lifestyle and dietary modifications. The National Kidney Foundation of America has highlighted the beneficial role of plant-based dietary patterns in enhancing insulin sensitivity, a critical factor in maintaining glycaemic control and reducing the risk and severity of diabetes mellitus (DM) [9]. According to Wathanavasin et al., a plant-based diet, rich in fruits and vegetables, provides alkali precursors, magnesium, and unsaturated fatty acids, may slow CKD progression by reducing dietary acid load and improving blood pressure control. In addition, these diets are typically higher in antioxidants and fiber, which may beneficially modulate gut microbiota composition and thereby attenuate systemic inflammation through the reduction of gut-derived uremic toxins [10]. Adherence to a plant-based dietary pattern, exemplified by the Mediterranean diet (MD), along with reduced consumption of processed foods, has been associated with improvements in key cardiometabolic risk factors and may contribute to better disease management [4]. The MD is characterized by a dietary pattern that emphasizes the consumption of cereals, fruits, vegetables, legumes, dairy products, fish, extra virgin olive oil, and moderate amounts of wine. Moreover, the MD is rich in bioactive compounds, such as polyphenols, which have been associated with numerous of potential health benefits [11].

The MD has been extensively investigated for its potential to improve key metabolic and cardiovascular risk factors that underline the development of DN, namely DM and hypertension. A systematic review by Esposito et al., synthesizing evidence from meta-analyses and randomized controlled trials, examined the role of the MD in individuals with DM as well as in those at high risk of developing the disease. Their findings indicated that adherence to the MD was consistently associated with improved glycemic control, reduced insulin resistance, and a lower prevalence of coronary heart disease, supporting its safety and effectiveness in the management of DM [12]. These metabolic effects are particularly relevant, as hyperglycemia and insulin resistance are central to the pathogenesis of DN. Complementing this evidence, Filippou et al. conducted a systematic review and meta-analysis to evaluate the effects of the MD on blood pressure. Their results showed that adherence to the MD was associated with favorable changes in both systolic and diastolic blood pressure, particularly in individuals with elevated baseline systolic levels (around 130 mmHg) and when the duration of follow-up was longer (on average 16 weeks) [13]. This suggests that the MD may be more effective in populations at higher cardiovascular risk and highlights the importance of sustained adherence to the dietary pattern. Taken together, these findings support the hypothesis that the MD may beneficially influence two major determinants of renal damage, namely poor glycemic control and elevated blood pressure. By improving these risk factors, the MD may indirectly contribute to the preservation of renal function and potentially play a role in the onset and progression of DN.

Despite substantial evidence supporting the MD in improving metabolic and cardiovascular outcomes, its direct impact on the onset and progression of DN remains inadequately defined. The complex interplay among hyperglycemia, hypertension, and renal dysfunction highlights the need for focused research to clarify whether dietary interventions such as the MD may influence the natural course of DN. Addressing this knowledge gap is important for informing clinical practice and guiding public health strategies aimed at the prevention and management of DN. The aim of this study was to examine the association between adherence to the MD and the onset and/or progression of DN.

## Methods

### Reporting framework and protocol

The present review was not prospectively registered in a pre-specified protocol database such as PROSPERO. Nevertheless, the methodology and reporting of this review adhered to the Preferred Reporting Items for Systematic Reviews and Meta-Analysis (PRISMA 2020) statement [14].

### Search strategy and eligibility criteria

**Figure 1.**
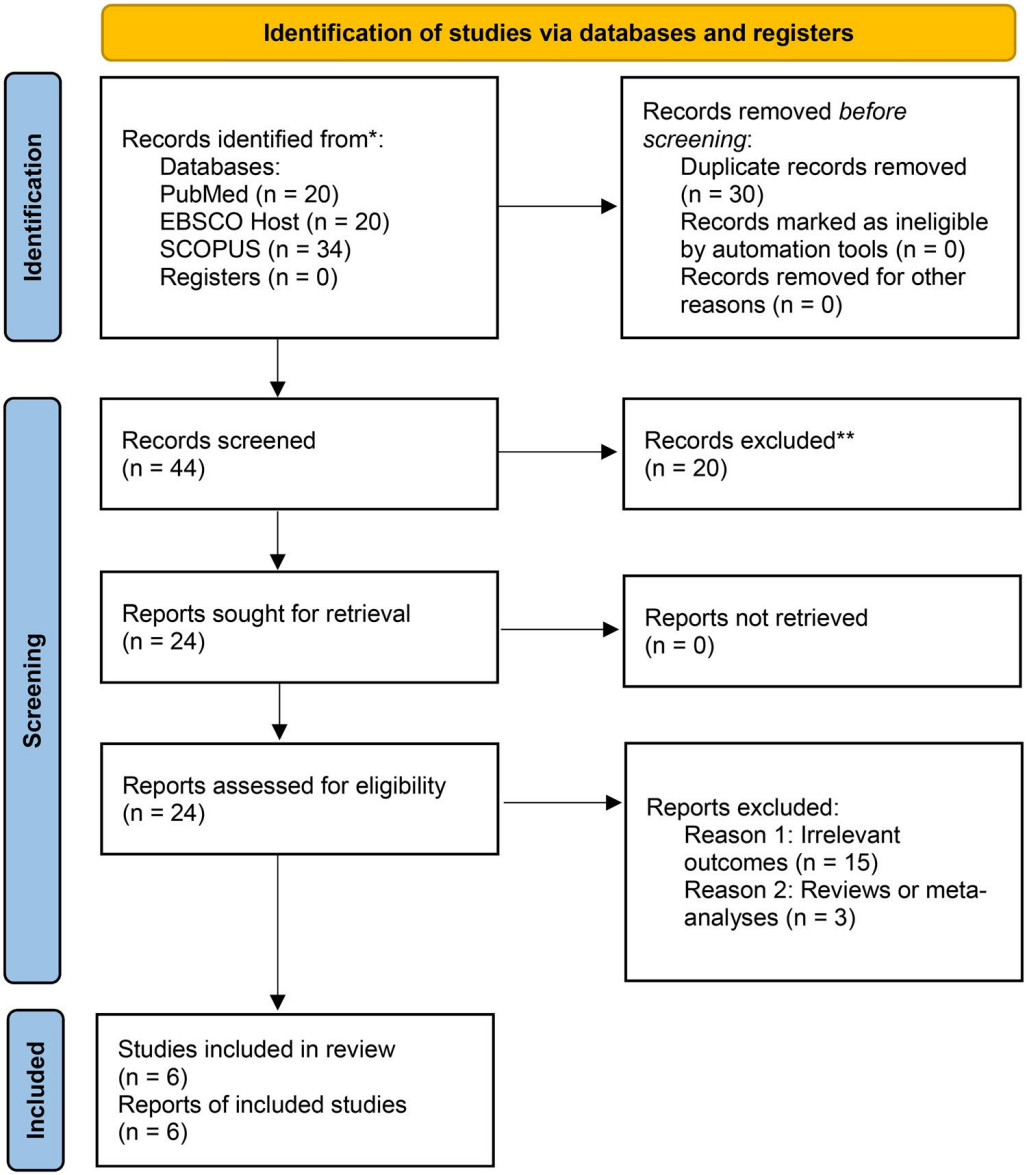
PRISMA 2020 flow diagram of study selection process. Source: Page et al. [17]. This work is licensed under CC BY 4.0. To view a copy of this license, visit https://creativecommons.org/licenses/by/4.0/

A systematic literature search was conducted using PubMed, EBSCO host and SCOPUS databases from inception to September 2025. The references cited in the selected articles were also searched manually. The study selection process is presented in Figure 1.

The search strategy combined terms related to the Mediterranean diet (e.g. ‘Mediterranean diet’) and diabetic nephropathy (e.g. ‘diabetic kidney disease’) using Boolean operators (AND, OR). The full search strategy used for this study is presented in Table 1.

**Table 1.** Search strategy across databases.

		Number of retrieved articles		
Search domains Keywords		PubMed	EBSCO Host	SCOPUS
Mediterranean diet – Exposure	1. Mediterranean diet	8,693	41,369	15,216
	2. Mediterranean diet program	6	324	1,106
	3. Mediterranean lifestyle	111	1,631	3,297
	4. Mediterranean diet plan	14	146	120
5. #1 OR #2 OR #3 OR #4		8,736	41,762	19,739
Diabetic Nephropathy – Outcome	6. Diabetic Nephropathy	23,035	68,339	22,220
	7. Diabetic Nephropathy risk	50	3,500	5,963
	8. Diabetic Nephropathy treatment	34	3,791	6,059
	9. Microvascular Complications	5,392	25,111	3,539
	10. Diabetic Kidney Disease	6,221	21,593	7,488
11. #6 OR #7 OR #8 OR #9 OR #10		32,318	105,377	38,701
12. #5 AND #11		20	20	34

Studies were considered eligible if they met the following criteria: Adults aged ≥ 18-year-old of any sex and ethnicity; Involved individuals with impaired glucose metabolism, including impaired fasting glucose (IFG), DM, or DN, with or without comorbidities; Examined adherence to the MD as the exposure or intervention; Reported outcomes related to the onset and/or progression of DN, including renal biomarkers such as albuminuria or estimated glomerular filtration rate (eGFR); Original primary studies of any methodological design included. Exclusion criteria included conference abstracts, dissertations, narrative reviews, case reports and studies not published in English.

### Study selection and data extraction

Data extraction was performed independently by two reviewers (IS and SC) using a predefined data extraction form. Any discrepancies were resolved through discussion with a third reviewer (MK). The outcomes of interest included DN incidence, DN progression, and kidney-related biomarkers such as ACR, BUN, creatinine, and eGFR. Only outcomes that were consistently reported and clinically relevant were extracted. Extracted data included: author, year, country, study design, follow-up duration, population characteristics, sample size, definition of MD adherence, renal outcomes assessed and main findings.

### Quality assessment/risk of bias

The assessment of the methodological quality and potential risk of bias of the included studies was performed by two independent reviewers (IS & SC). Any disagreement was resolved by discussion with a third author (ΜΚ). Non-clinical studies were evaluated using the Newcastle-Ottawa quality assessment scale (NOS) for cohort studies and the modified version for cross-sectional studies [14]. For cohort studies, the maximum final score is 9, while studies that achieve a score ≥7 are of high methodological quality. For cross sectional studies, 10 stars is the maximum score and studies with a score ≥8 are of high methodological quality [15]. The Jadad score was used for the assessment of clinical studies based on the randomization, the blinding and the participants’ dropout. The final score ranges from 0 to 5 points and a Jadad score ≥3 is considered ‘high quality’ whereas a Jadad score ≤2 is considered of ‘low’ quality for Jadad et al. [16]. This assessment focused on key domains such as selection bias, comparability/confounding, and outcome assessment.

### Synthesis of results

Due to substantial heterogeneity in study design, populations, definitions of MD adherence, and outcome measures, a quantitative synthesis (meta-analysis) was not considered appropriate. Therefore, findings were synthesized narratively. Studies were grouped according to whether they examined the onset of DN or progression in individuals with established disease. Effect measures included odds ratios (Ors), hazard ratios (HRs), and percentage risk reductions, as reported by the included studies. No pooled estimates were calculated due to study heterogeneity.

No data conversions or statistical transformations were required for the synthesis. Data were extracted and presented as reported in the original studies. In cases of missing data, no imputation was performed, and only available data were included.

## Results

### Search results

Following the database search and removal of duplicates, records were screened according to the predefined eligibility criteria. Several full-text articles were excluded after eligibility assessment due to not meeting the inclusion criteria. The main reasons for exclusion included irrelevant outcomes, non-adult populations, lack of assessment of adherence to the MD, or inappropriate study design. Six studies were deemed eligible for inclusion in the present review. The study selection process is presented in Figure 1.

### Characteristics of included studies

The included studies involved healthy individuals, as well as individuals with impaired glucose metabolism, DM or DN. In all studies, adherence to the MD was the main exposure of interest. Most studies were conducted in Iran (Moradi et al.; Jayedi et al.; Noori et al.; Ghaemi et al.), with one study conducted in the United Kingdom (Qu et al.) and one in Spain (Dia Lopez et al.). The main characteristics and findings of the included studies are presented in Table 2.

**Table 2.** Summary of included studies investigating the association between adherence to the Mediterranean diet (MD) and diabetic nephropathy (DN).

		Main characteristics				MD adherence assessment tool		
Study/country	Study design	Participants characteristics	Sample size	Mean age (years)	Follow-up duration		Main renal outcomes / results	Study quality results
Qu et al. [17]/United Kingdom,	Prospective cohort	Individuals with hyperglycemia free of microvascular complications at baseline (T2DM and non-T2DM hyperglycemia)	33,441	NR	12.3 years	Alternate Mediterranean diet (AMED) score	Higher MD adherence associated with lower DKD risk: HR= 0.79 (95% CI: 0.67 -–0.94); stronger association in T2DM: HR = 0.64 (95% CI: 0.50–0.83) in individuals with T2DM	9/9* (NOS)
Moradi et al. [22]/Iran	Cross-sectional	Patients with DN	270	65.9 ± 9.7 years	NR	MD based on traditional MedDiet characteristics using FFQ	No significant association between MD adherence and renal biomarkers [Cr (*p* = 0.343), BUN (*p* = 0.680), eGFR (*p* = 0.813)].	7/9* (NOS)
Jayedi et al. [18]/Iran	Case-control	Women with T2DM with DN (ACR ≥ 30mg/g) and without DN.	210	55.3 ± 7.0 years (DN); 55.4 ± 7.1 years (without DN)	NR	MD score (Trichopoulou method) using validated FFQ	Moderate and high adherence to the MD were associated with 62% [OR = 0.38 (95% CI: 0.20–0.73)] and 86% [OR = 0.14 (95% CI: 0.06–0.33), (*p* < 0.001)] lower odds of DN, respectively.	9/9* (NOS)
Noori et al. [19]/Iran	Case-control	Women with T2DM with DN (ACR ≥ 30mg/g) and without DN.	210	30–65 years	NR	Modified MD score (Trichopoulou method) using FFQ	Higher adherence to the MD was associated with a lower likelihood of DN in both crude [OR = 0.272 (95% CI: 0.154–0.481)] and adjusted models [OR = 0.239 (95% CI: 0.128–0.447); *p* = 0.001].	8/9* (NOS)
Ghaemi et al. [20]/Iran	Longitudinal study	Adults with T1DM and T2DM	22,187	T1DM: 50.7; T2DM: 59.9 years	February 2016–March 2020	14-item MD adherence questionnaire	Lower nephropathy incidence in T1DM: OR= 0.42, (95% CI: 0.30–0.58), *p* < 0.001); T2DM: OR = 0.88, (95% CI: 0.80–0.96), *p* = 0.007).	8/9* (NOS)
Díaz-López et al. [21]/Spain	Post-hoc analysis Of randomized trial	T2DM participants free of microvascular complications and at high cardiovascular risk	3,614	55–80 years	Median 6.0 years (October 2003 to January 2009)	MD intervention with FFQ-based adherence assessment	No significant association between MD interventions and nephropathy incidence: HR = 1.15 (95% CI: 0.79–1.67) for MedDiet + EVOO & 1.06 (95% CI: 0.72–1.58) for MedDiet + Nuts.	4/5** (Jadad)

**Abbreviations:** MD: Mediterranean diet; AMED: alternate Mediterranean diet score; DN: Diabetic nephropathy; DKD: Diabetic Kidney Disease; HR: Hazard Ratio; OR: Odds Ratio; CI: Confidence Interval; Cr: Creatinine; BUN: Blood urea nitrogen; eGFR: estimated Glomerular Filtration Rate; FFQ: Food Frequency Questionnaire; ACR: Albumin-to-Creatinine Ratio; T1DM: Type 1 Diabetes Mellitus; T2DM: Type 2 Diabetes Mellitus; MedDiet + EVOO: Mediterranean diet supplemented with Extra Virgin Olive Oil; MedDiet + Nuts: Mediterranean diet supplemented with Nuts.

* NOS: Newcastle Ottawa Scale (Nayebirad et al.).

** Jadad Scale (Jadad et al.).

### Risk of bias assessment

Regarding the methodological quality, the studies by Qu et al. and Jayedi et al. received the maximum score of 9 stars on the NOS, while the study by Ghaemi et al. and Noori et al. was rated 8 stars. The cross-sectional study by Moradi et al. received 7 stars and was considered of good methodological quality. The post-hoc analysis by Díaz-López et al. achieved 4 stars, based on the Jadad scale. Overall, the studies included were of moderate to high methodological quality. Table 3 summarizes the overall methodological quality of the included studies, while detailed domain-level risk of bias assessments are presented in Supplementary Figures 1-4.

**Table 3.** Limitations & risk of bias of the included studies.

Study	Heterogeneity/population	Selection criteria/bias	Confounding	Outcome assessment	Follow-up	Assessment of MD adherence
Qu et al. [17]	Population restricted to a specific cohort;	Cohort-based recruitment may limit representativeness of the broader diabetic population.	Despite adjustment, factors such as comorbidities, medications, physical activity, and family history of vascular disease may influence both diet and renal risk.	Renal outcomes assessed using specific definitions/markers for DN.	Outcome timing and progression pathways may still vary across participants.	Slightly modified tool (AMED) compared to MED score.
Moradi et al. [22]	Small sample, specific clinical group & geographic setting.	Cross-sectional recruitment.	Other diseases, medication use.	Renal functions assessed at one time point.	No follow-up.	Through dietary self-report (recall and measurement bias).
Jayedi et al. [18]	Small, sex-specific sample & one geographic context.	Vulnerability to selection of case and controls.	Residual confounding remains possible despite adjustment.	Based on specific criteria (e.g. ACR).	Retrospective nature limits temporal interpretation.	Assessment tool based on Trichopoulou et al.
Noori et al. [19]	Mixed population including DM & DN; sex-restricted and region-specific sample.	Case-control sampling may introduce selection bias.	Residual confounding remains possible despite adjustment.	Included renal biomarkers such as ACR, BUN & creatinine.	Limited ability to assess long-term progression because of design.	Derived from questionnaire-based scoring.
Ghaemi et al. [20]	Heterogenous diabetic population (type 1 & 2).	Limited generalizability (population-specific cohort).	Residual confounding possible from medications, complication and baseline disease severity.	Variation in the classification of kidney disease (not clearly defined as DN).	Follow-up was available; however, it was the only longitudinal study.	Derived from dietary pattern assessment.
Díaz-López et al. [21]	Population at high cardiovascular risk.	Post-hoc subgroup analysis may introduce analytical limitations.	Post-hoc nature & competing clinical factors still require caution.	Nephropathy incidence remained limited.	Median follow-up reported, but nephropathy cases were relatively few.	Intervention-based MD assessment.

#### Definitions of MD adherence and DN outcomes

The studies included used different tools and scoring systems to assess adherence to the MD. Most studies evaluated MD adherence using validated Food Frequency Questionnaires (FFQs) combined with MD scoring systems based on Trichopoulou method, while Qu et al. used the Alternate MD (AMED) score and Ghaemi et al. used a 14-item MD adherence questionnaire. In most studies, higher scores reflected greater adherence to the MD dietary pattern.

Definitions of DN and DN progression also varied among the included studies. Most studies defined DN based on albumin-to-creatinine ratio (ACR) thresholds, estimated glomerular filtration rate (eGFR), or clinically diagnosed diabetic kidney disease (DKD). Specifically, Jayedi et al. and Noori et al. defined DN as ACR ≥30 mg/g, while Qu et al. identified DKD using ICD-10 diagnostic criteria within the UK Biobank cohort. Díaz-López et al. evaluated incident nephropathy using repeated measurements of albuminuria and impaired renal function during follow-up. Due to methodological heterogeneity across studies, definitions and assessment methods for both MD adherence and DN outcomes are summarized in Supplementary Table 1.

#### Studies on DN incidence

Five of the included studies investigated the association between adherence to MD and the onset of DN. Qu et al., in a prospective cohort study among individuals with hyperglycemia and Type 2 Diabetes Mellitus (T2DM), reported that higher adherence to the MD was associated with a lower risk of DKD, with an estimated 21% risk reduction in the overall cohort [HR = 0.79 (95% CI: 0.67–0.94)] and a stronger protective association among individuals with established T2DM [HR = 0.64 (95% CI: 0.50–0.83)] [17]. Jayedi et al., in a case-control study among women with T2DM, reported that moderate and high adherence to the MD were associated with 62% [OR = 0.38 (95% CI: 0.20–0.73)] and 86% [OR = 0.14 (95% CI: 0.06–0.33)], lower odds of DN, respectively, compared with low adherence (*p* < 0.001) [18]. Similarly, Noori et al., in a case-control among women with diabetes and/or DN, observed that higher adherence to the MD was associated with a lower likelihood of DN in both crude [OR = 0.272 (95% CI: 0.154–0.481)] and adjusted models [OR = 0.239 (95% CI: 0.128–0.447); *p* = 0.001]. Greater consumption of key MD components, such as legumes [OR: 0.156; 95% CI: 0.083–0.292; *p* = 0.001), vegetables [OR: 0.273; 95% CI: 0.149–0.501; *p* = 0.001), fruits [OR: 0.179; 95% CI: 0.093–0.347; *p* = 0.001), and fish [OR: 0.459; 95% CI: 0.254–0.827; *p* = 0.001} was also associated with reduced odds of DN [19]. Ghaemi et al., in a longitudinal cohort among individuals with Type 1 Diabetes Mellitus (T1DM) and T2DM, reported that adherence to the MD was associated with a lower incidence of nephropathy in both T1DM [OR= 0.42, (95% CI: 0.30–0.58); *p* < 0.001]; and T2DM [OR = 0.88, (95% CI: 0.80–0.96); *p* = 0.007] [20].

In contrast, Díaz-López et al. based on a post-hoc analysis of the PREDIMED randomized controlled trial, among individuals with T2DM at high cardiovascular risk, reported no significant association between MD interventions and nephropathy incidence [HR = 1.15 (95% CI: 0.79–1.67) for MedDiet + EVOO and 1.06 (95% CI: 0.72–1.58)] for MedDiet + Nuts [21].

#### Studies on DN progression

Only two of the included studies examined the association between adherence to the MD and the progression of DN among individuals with established disease. Evidence from Moradi et al., in a cross-sectional analysis, reports no significant association between MD and improvements in renal biomarkers, including creatinine [Cr (*p* = 0.343), blood urea nitrogen [BUN (*p* = 0.680)], and eGFR (*p* = 0.813), among patients with DN [22]. On the contrary, Noori et al., in case-control study among women with T2DM and DN, reported that higher adherence to the MD was associated with lower BUN levels (*p* = 0.039), while reductions in albumin-to-creatinine ratio (ACR) and serum creatinine did not reach statistical significance [19].

The main characteristics and findings of the included studies are presented in Table 2.

## Discussion

### Adherence to the MD and its effect on DN

The primary objective of this systematic review was to evaluate the association between adherence to the MD and the incidence and progression of DN. Across the included studies, higher adherence to the MD was generally defined using validated FFQs and MD scoring systems, including AMED score and Trichopoulou-based MD indices, where higher scores reflected greater adherence to the dietary pattern. Common MD components across the included studies incorporated higher consumption of fruits, vegetables, legumes, whole grains, fish, nuts, and olive oil, alongside lower consumption of red and processed meat. Overall, the findings suggest that greater adherence to the MD may be associated with a reduced risk of incident DN in individuals with diabetes, although evidence regarding disease progression remains limited and inconsistent. A quantitative meta-analysis was not performed due to substantial methodological heterogeneity among the included studies, including differences in study design, MD adherence assessment tools, DN definitions, renal outcomes measures, and reported effect estimates.

Overall, the available evidence suggests a potential beneficial association between greater adherence to the MD and reduced risk of incident DN in individuals with diabetes. However, evidence regarding the progression of established disease remains limited and inconsistent.

### Potential mechanisms of the MD

Potential mechanisms underlying the beneficial association between the MD and DN may involve improvements in glycemic control, blood pressure regulation, body weight, and systemic inflammation [10,23]. Whole grains and the high dietary fiber content of the MD may contribute to improved insulin sensitivity, delayed glucose absorption, and lower postprandial glycemic excursions. In addition, the MD is rich in monounsaturated fatty acids, antioxidants, and anti-inflammatory compounds, which may reduce oxidative stress and endothelial dysfunction [10,23]. Evidence from the meta-analysis by Wu et al. demonstrated that adherence to the MD was associated with significant reductions in HbA1c [MD: −0.307% (95% CI: −0.451 to −0.163)], fasting plasma glucose [MD: −0.845 mmol/L, 95% CI: −1.307 to −0.384), and BMI (MD −0.828 kg/m^2^, 95% CI: −1.4 to −0.256) among individuals with T2DM. Additional cardiometabolic improvements included reductions in LDL-C [MD: −8.060 mg/dL, 95% CI: −14.213 to −1.907], systolic blood pressure (SBP) [MD −5.130 mmHg, 95% CI: −10.877 to 0.617), and diastolic blood pressure (DBP) [MD −2.008 mmHg, 95% CI: −3.027 to −0.989]. These metabolic and hemodynamic improvements may contribute to reduced glomerular hyperfiltration and slower progression of DN [23].

### Studies on DN incidence

Five of the included studies investigating the incidence of DN, reported findings suggesting a favorable association between adherence to the MD and renal outcomes, which is in line with previous observational studies reporting beneficial associations between the MD and kidney function across different populations [17–21]. Previous evidence from non-diseased populations further reinforces the potential renal benefits of the MD. In the ATTICA study, greater adherence to the MD was associated with improved renal function parameters among healthy adults, suggesting a potential protective role even in early, non-clinical stages of kidney dysfunction [24]. Similarly, the 3 L study in Greek adolescents demonstrated that higher adherence to the MD was linked to lower levels of albuminuria, an early marker of renal damage, indicating that beneficial associations may already be present from adolescence [25]. These findings are in line with the results of the present review, which also observed a favorable association between higher MD adherence and reduced risk of DN, supporting the hypothesis that the protective effects of the MD may extend across different age groups and clinical conditions, including both early kidney dysfunction and overt diabetic kidney disease.

Notably, while the prospective design of the included studies, sample size, and adjustment for confounding factors strengthen the internal validity of the findings, the observational nature of the study should be considered when interpreting the results.

In contrast, from a clinical perspective, Díaz-López et al., in a post-hoc analysis of a randomized controlled trial, reported no significant protective effect of the MD on DN incidence, although beneficial effects were observed for other microvascular outcomes such as diabetic retinopathy (*p* > 0.05) [21]. These findings may reflect differences in outcome sensitivity or underlying pathophysiological mechanisms, although alternative explanations related to study design and comparator dietary patterns cannot be excluded. The post-hoc nature of the study, despite derives from a randomized controlled trial, was not originally designed to specifically evaluate DN outcomes. Therefore, the absence of significant findings in these studies may reflect methodological limitations rather than absence of a true effect.

Taken together, the available evidence suggests a potential beneficial association between MD adherence and DN risk; however given the methodological limitations of most studies and the heterogeneity across populations and methodologies, these findings should be interpreted with caution.

### Studies on DN progression

Only two of the included studies examined the effect of the MD in the progression of the disease. Moradi et al. in a cross-sectional study among patients with established DN, found no statistically significant association between MD adherence and renal function (*p* > 0.05) [22]. These null findings may be attributed to the limitations of the study design, which does not allow causal inference, as well as by participant characteristics, including older age, high baseline cardiovascular risk, and the presence of comorbid conditions such as metabolic syndrome.

Evidence on the progression of DN remains inadequately explored; however, Noori et al., in a case-control study including women with diabetes and/or DN, provided some insight into this aspect. The study reported that higher adherence to the MD was associated with improved kidney-related markers, including ACR, BUN, and serum creatinine [19]. These findings suggest a potentially beneficial role of the MD in the clinical management of DN, possibly through improvements in metabolic control and reduction of renal burden. Nevertheless, given the observational design and the limited number of studies focusing specifically on disease progression, these results do not provide certainty.

Importantly, the available evidence on patients with established DN is limited, and current findings are insufficient to determine whether adherence to MD influences disease progression. Further well-designed prospective studies and randomized controlled trials with longer follow-up are required to better clarify the role of the MD in the progression of DN.

### Potential mechanisms & dietary implications

Current KDOQI (Kidney Disease Outcomes Quality Initiative) guidelines and recent KDIGO recommendations on protein intake in individuals with chronic kidney disease (CKD) emphasize that at least 50% of the daily protein intake no longer needs to be derived from high biological value animal sources, reflecting the growing recognition of the potential benefits of plant-based proteins [5,26]. This shift highlights the importance of dietary quality rather than a strict focus on protein sources. Importantly, excessive restriction of potassium and phosphorus, which is sometimes implemented in the management of kidney disease, may lead to reduced dietary fiber intake. Lower fiber intake has been associated with alterations in gut microbiota composition, increased production of gut-derived uremic toxins, disruption of the intestinal epithelial barrier, and increased systemic inflammation [27,28]. In line with these observations, Carrero et al. reviewed the evidence on plant-based dietary patterns, including MD, and reported several potential benefits for individuals with kidney disease. These include associations with preservation of eGFR, improved insulin sensitivity, reduced risk of sarcopenia, and favorable effects on bone health, factors particularly relevant in slowing kidney failure progression [28]. Taken together, combined with the current evidence, it supports the notion that plant-based dietary patterns, such as MD, may contribute to renal protection not only through the modulation of traditional risk factors, including glycemic control and blood pressure, but also by exerting favorable effects on gut health, inflammation, and metabolic homeostasis. However, the evidence in these emerging areas remains limited, and further well-designed studies are required to clarify these mechanisms. Consequently, while current guidelines and available evidence support the inclusion of plant-based dietary patterns as part of the management of CKD, the role of the MD in DN should be interpreted within the context of a comprehensive multifactorial treatment approach.

### Generalizability & clinical context

An important limitation of the available evidence relates to its generalizability. Most of the included studies were conducted in specific geographic regions, primarily Iran, with only one study based on the UK Biobank and one conducted in Spain. Differences in cultural and dietary patterns across these regions may influence both adherence to and interpretation of the MD. In particular, the application of the MD in non-Mediterranean settings, such as Iran, may differ substantially from its traditional form in Southern Europe, potentially affecting the consistency and comparability of findings across studies.

Furthermore, current clinical guidelines, such as those from KDIGO, emphasize a comprehensive approach to the management of diabetic kidney disease that includes both pharmacological and lifestyle interventions [5]. In this context, pharmacological therapies such as metformin, SGLT2 inhibitors, GLP-1 receptor agonists and DPP-4 inhibitors have demonstrated significant benefits in slowing the progression of diabetes and kidney disease, respectively, and reducing overall cardiovascular risk [5].

Within this evolving therapeutic landscape, dietary patterns such as the MD may be considered as part of a broader, multifactorial strategy rather than a standalone intervention. While the MD may contribute to improved metabolic control and potentially favorable renal outcomes, its role should be interpreted alongside established pharmacological treatments and individualized patient care. Further research is needed to clarify how dietary interventions can be optimally integrated into contemporary standards of diabetes and chronic kidney disease management.

### Strengths & limitations

This review has several strengths, although these should be considered alongside its limitations. To our knowledge, it represents one of the first comprehensive synthesis specifically focusing on the association between adherence to the MD on the onset and/or management of DN. Most of the included studies were of moderate to high methodological quality; however, important limitations related to study design and potential residual confounding should be considered. The number of available studies was limited, and most were non-clinical in nature, restricting the ability to establish causal inferences between MD adherence and renal outcomes. The study populations were heterogeneous in terms of age, comorbidities, sample size, inclusion criteria and geographic background, which may influence the observed associations and limit generalizability. Moreover, several important confounding factors associated with the incidence and progression of DN, including baseline blood pressure, glycemic control (HbA1c), smoking status, comorbidities, medication use, physical activity, and family history of vascular disease, may not have been fully accounted across the included studies. Concurrent pharmacological treatments, such as renin-angiotensin system (RAS) blockers, sodium-glucose cotransporter-2 (SGLT2) inhibitors, and finerenone, may independently influence renal outcomes beyond adherence to the MD. Variability in the assessment of renal outcomes, as well as differences in the tools used to evaluate adherence to the MD, including different scoring systems and geographic adaptations, further reduced comparability across studies. In addition, most included studies were observational and heterogeneous in design (cohort, case-control, and cross-sectional), increasing susceptibility to selection bias and residual confounding. Collectively, these methodological limitations reduce the overall certainty of the findings and highlight the need for future studies with standardized methodologies and higher-quality longitudinal designs.

### Implications for future research

Consequently, there is a clear need for future research, particularly well-designed prospective and interventional clinical studies, to more rigorously investigate the role of the MD in the progression of DN among patients with established disease. Such studies are essential to overcome the current methodological limitations, reduce heterogeneity across study designs, and provide more definitive evidence regarding the potential impact of dietary adherence on clinically meaningful renal outcomes.

## Conclusion

Overall, this review contributes to the existing body of literature suggesting a potential association between adherence to MD, as a non-pharmacological intervention, in the onset and preservation of DN, thereby reinforcing its relevance in clinical nutrition and public health strategies. However, the key challenge lies in producing robust evidence that is applicable in routine clinical practice, while keeping patient-centered care at the forefront. This review highlights a substantial gap in the current literature regarding the impact of plant-based dietary patterns on one of the most serious non-communicable diseases, diabetic nephropathy, and underscores the need for further well-designed studies in this area.

## Supplementary Material

Supplementary Material Risk of Bias.docx

Supplementary Table 1.docx

## Data Availability

The authors confirm that the data supporting the findings of this study are available within the article and its supplementary materials.

## Registration

This review was not registered.
