# Supplementary material for: Adherence to the Mediterranean diet and its effects on the onset and progression of diabetic nephropathy: a systematic review
Source: Ren Fail. 2026 Jul 27;48(1):2685372. doi: 10.1080/0886022X.2026.2685372 (PMC13410535; doi:10.1080/0886022X.2026.2685372)
Supplement: Supplementary Material Risk of Bias.docx [file IRNF_A_2685372_SM2715.docx]

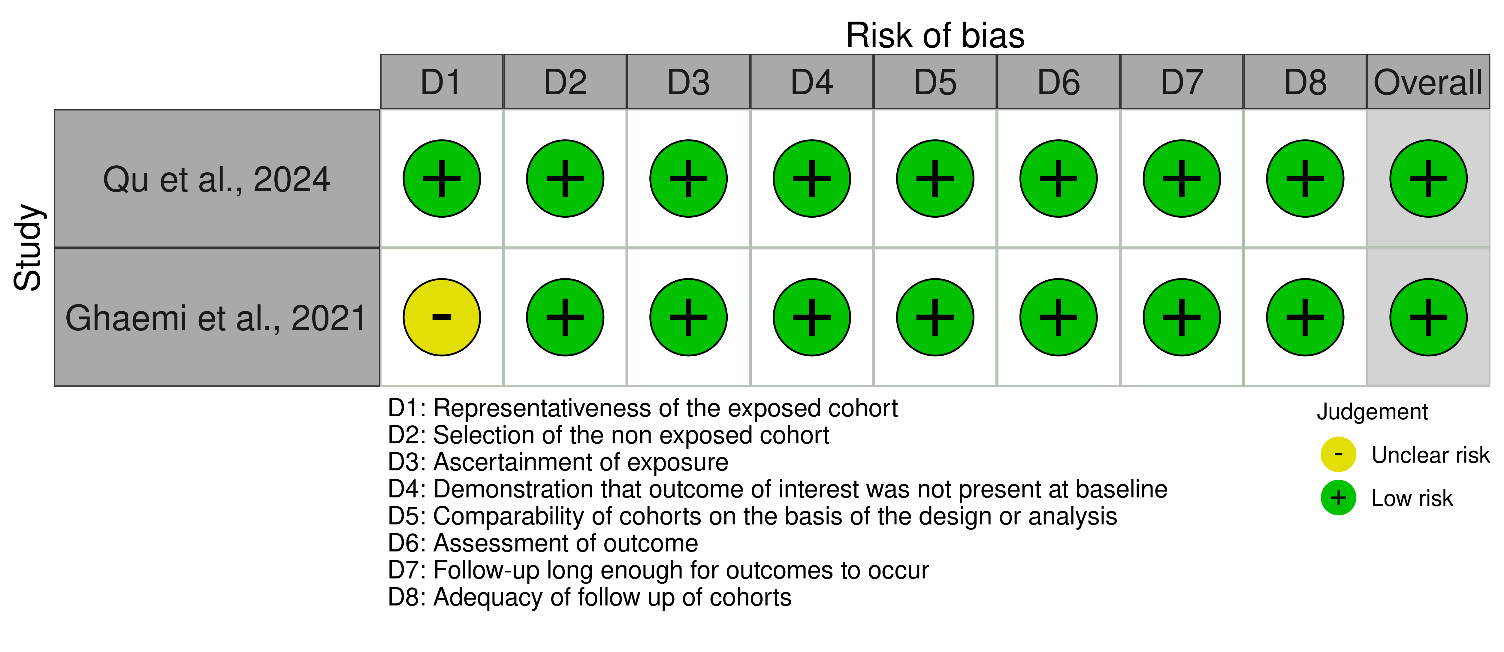
**Supplementary Figure 1.** Detailed Newcastle–Ottawa Scale (NOS) assessment of risk of bias for cohort studies.

**Supplementary Figure 2.** Detailed Newcastle–Ottawa Scale (NOS) assessment of risk of bias for case–control studies.


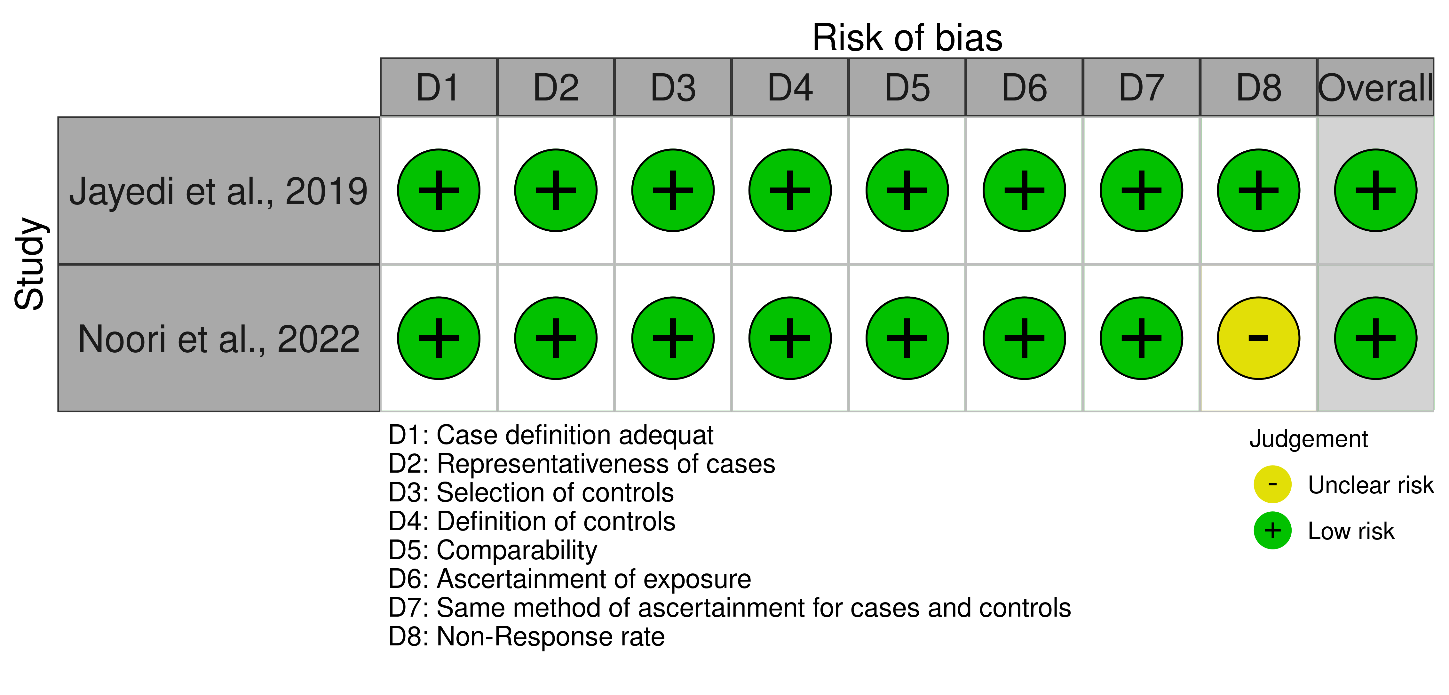


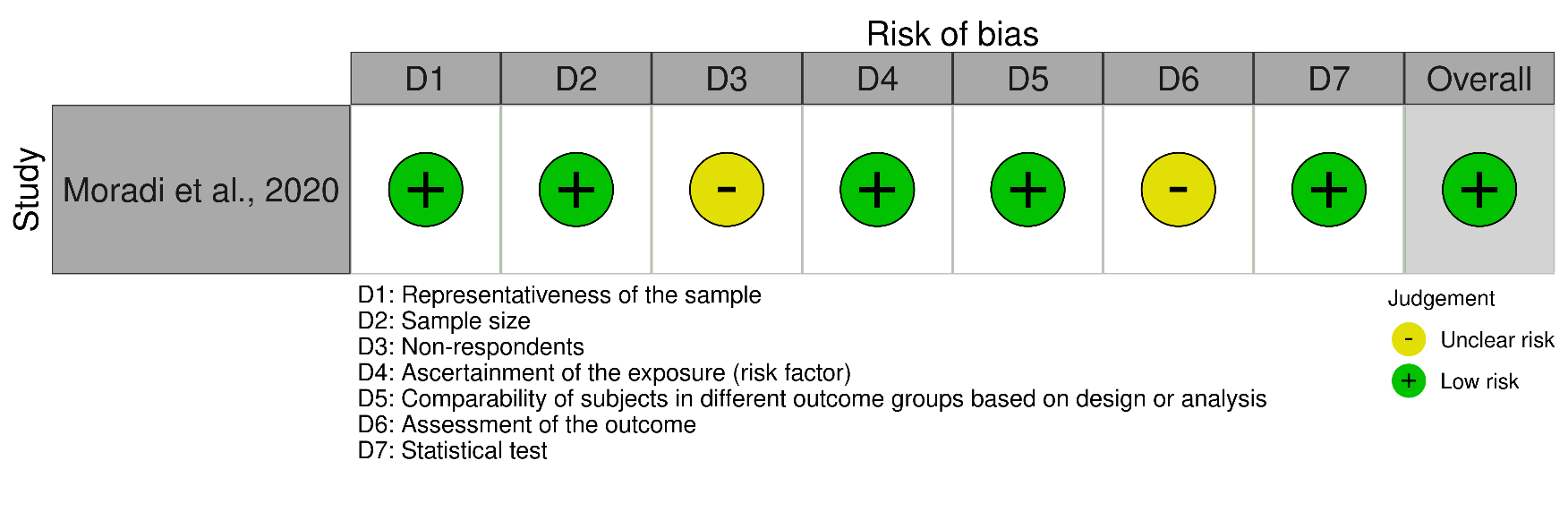
**Supplementary Figure 3.** Detailed Newcastle–Ottawa Scale (NOS) assessment of risk of bias for the cross-sectional study.


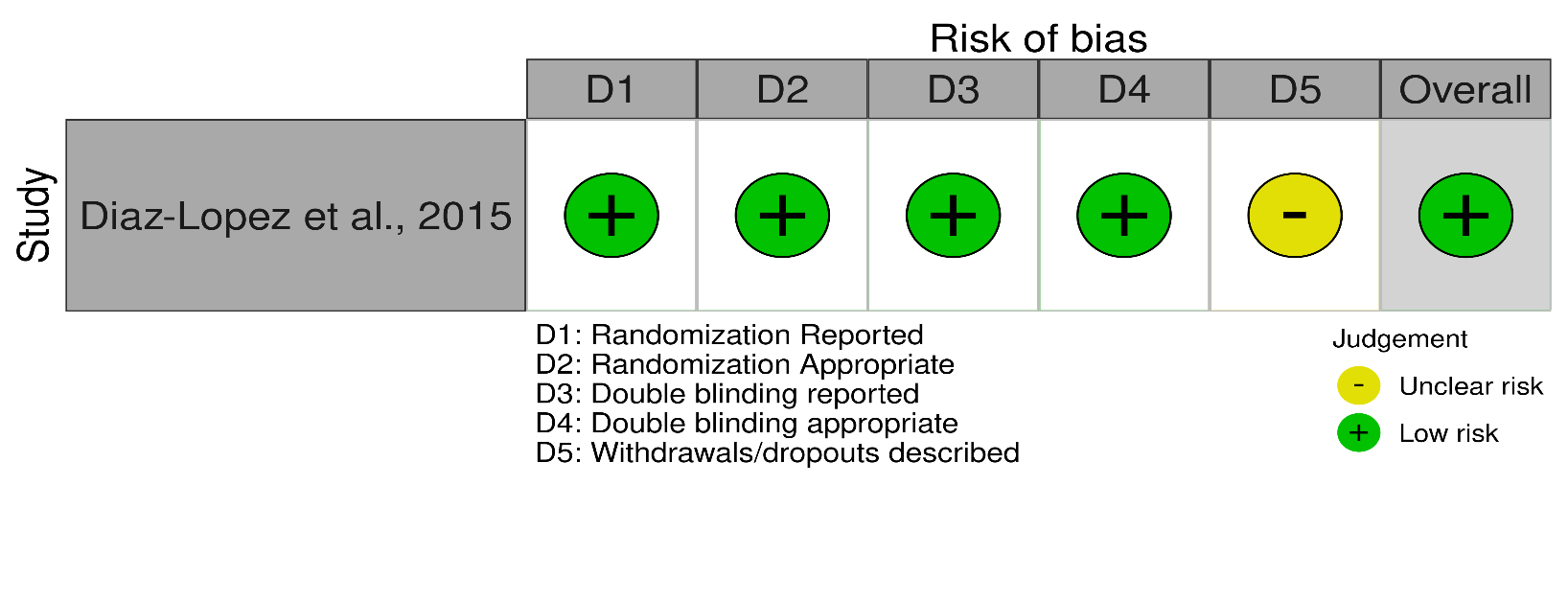
**Supplementary Figure 4.** Detailed Jadad Scale assessment of risk of bias for the Post-hoc analysis of a randomized controlled trial.
