# Supplementary material for: Adherence to the Mediterranean diet and its effects on the onset and progression of diabetic nephropathy: a systematic review
Source: Ren Fail. 2026 Jul 27;48(1):2685372. doi: 10.1080/0886022X.2026.2685372 (PMC13410535; doi:10.1080/0886022X.2026.2685372)
Supplement: Supplementary Table 1.docx [file IRNF_A_2685372_SM2714.docx]

**Supplementary Table 1.** Definition of MD adherence & DN outcomes.

| Study | MD adherence assessment | Definition of DN/DN progression |
| --- | --- | --- |
| Qu et al. (2024) | AMED score | ICD-10 defined CKD |
| Moradi et al.  (2020) | MD score based on Trichopoulou method using FFQ | DN stage 1-2 patients with proteinuria and impaired renal biomarkers. |
| Jayedi et al.  (2019) | MD score based on Trichopoulou method using FFQ | DN defined as ACR ≥30 mg/g, |
| Ghaemi et al.  (2021) | 14-item MD adherence questionnaire | Incident nephropathy from national diabetes registry. |
| Noori et al.  (2022) | Modified MD score based on Trichopoulou method using FFQ | DN defined as ACR ≥30 mg/g, |
| Diaz-Lopez et al.  (2015) | FFQ-based MD intervention | Albuminuria/eGFR decline |

**Abbreviations:** MD: Mediterranean Diet; AMED: Alternate Mediterranean Diet Score; DN: Diabetic Nephropathy; CKD; Chronic Kidney Disease; ICD-10: International Classification of Diseases, 10^th^ Revision; FFQ: Food Frequency Questionnaire; ACR: Albumin-to-Creatinine Ratio; eGFR: estimated Glomerular Filtration Rate.
